# Smoking and Mental Illness: Prevalence, Patterns and Correlates of Smoking and Smoking Cessation among Psychiatric Patients

**DOI:** 10.3390/ijerph17155571

**Published:** 2020-08-01

**Authors:** P. V. Asharani, Vanessa Ai Ling Seet, Edimansyah Abdin, Fiona Devi Siva Kumar, Peizhi Wang, Kumarasan Roystonn, Ying Ying Lee, Laxman Cetty, Wen Lin Teh, Swapna Verma, Yee Ming Mok, Daniel Shuen Sheng Fung, Siow Ann Chong, Mythily Subramaniam

**Affiliations:** 1 Research Division, Institute of Mental Health, Singapore 539747, Singapore; Ai_Ling_SEET@imh.com.sg (V.A.L.S.); edimansyah_abdin@imh.com.sg (E.A.); Fiona_Devi_SIVA_KUMAR@imh.com.sg (F.D.S.K.); peizhi_wang@imh.com.sg (P.W.); K_ROYSTONN@imh.com.sg (K.R.); Ying_Ying_LEE@imh.com.sg (Y.Y.L.); Laxman_CETTY@imh.com.sg (L.C.); Wen_Lin_TEH@imh.com.sg (W.L.T.); siow_ann_chong@imh.com.sg (S.A.C.); mythily@imh.com.sg (M.S.); 2 Early Psychosis Intervention Programme, Institute of Mental Health, Singapore 539747, Singapore; swapna_verma@imh.com.sg; 3 Department of Mood and Anxiety, Institute of Mental Health, Singapore 539747, Singapore; yee_ming_mok@imh.com.sg; 4 Developmental Psychiatry, Institute of Mental Health, Singapore 539747, Singapore; Daniel_Fung@imh.com.sg

**Keywords:** smoking cessation, electronic nicotine delivery systems, smokers, past smokers

## Abstract

This study aims to understand (a) the prevalence and correlates of smoking in a psychiatric population, (b) factors that encourage smoking cessation, and (c) awareness towards cessation programmes. This study captured data (*n* = 380) through a modified version of the Global Adult Tobacco Survey (GATS). A descriptive analysis of the data was performed. The prevalence of smoking was 39.5% (*n* = 150) and 52.3% of the smokers were dependent on nicotine. More than half of the smokers had made at least one attempt to quit in the past 12 months and 56% reported no immediate plans to quit smoking. The awareness towards institutional smoking cessation programmes was fair (44%), with 49.7% of smokers having indicated that they were willing to use the service upon referral. Smokers endorsed that increasing the cost of cigarettes, restricting availability, and increasing knowledge of health harms could encourage smoking cessation. Past smokers reported that self-determination/willpower followed by substitution of smoking with other types of foods and drinks were factors that helped them achieve successful cessation. Given that the readiness to quit and awareness towards cessation programmes are low among the smokers, concerted efforts through educational programmes and policy changes are crucial to achieve successful cessation.

## 1. Introduction

Smoking is one of the leading causes of mortality and morbidity worldwide, accounting for 8 million deaths globally; with more than 7 million deaths directly related to tobacco use [1]. The mortality rate is three times higher in smokers and they die an average of 10 years earlier than non-smokers [2]. Tobacco consumption also increases the risk of lung diseases, stroke, cardiovascular diseases, and cancer [3]. The risk of head and neck cancer and stroke are 10 times and two to four times higher, respectively, in smokers than in non-smokers [3,4]. A global treaty for the control and prevention of tobacco use (World Health Organisation Framework Convention on Tobacco Control) [5], was initialised with the intent to reduce the global prevalence of tobacco use by 30% in 2025. The prevalence of smoking in Western countries decreased in the recent years whereas, the smoking rates in Asia showed no signs of improvement with China, India and Indonesia showing the highest number of smokers and thus higher disease burden [6,7,8]. 

In Singapore, the prevalence of daily smoking had dropped from 14.3% in 2010 to 12% in 2017 among those aged 18 to 69 years old [9,10]. In 2012, a national level epidemiological study among individuals aged 18 and above showed that 16% of the resident population were current smokers [11] with 4.5% dependent on nicotine. A recent national survey using the same methodology showed no change in the prevalence of smoking in Singapore (16.1%) over the years [12], despite the ongoing awareness programmes. In addition, the prevalence of nicotine dependence (ND) decreased from 4.5% to 3.3% [12]. The National Registry of Diseases in Singapore [13] registered higher rates of smoking among those with cardiovascular diseases (43.2%), stroke (36.4%), and cancer. 

There are certain risk groups who are more prone to tobacco use and ND. Previous studies have noted strong associations between ND and psychiatric diseases [14,15,16]. When compared to the general population, those with mental illness had 2 to 3.2 times higher risk of smoking and 25% less chance of quitting [17,18]. Previous studies have shown that the prevalence of smoking is higher in individuals with major depressive disorder, schizophrenia, and bipolar disorder [19,20]. This is hypothesised to be due to a combination genetic/biological, social and psychological factors. Genetic predisposition towards smoking, poor coping strategies, smoking as self-medication to cope with the symptoms of mental illness, or as a social reinforcement where smoking is a social activity/culture in mental health/rehabilitation facilities, and the higher severity of withdrawal symptoms [21,22] have all been implicated. Nicotine modulates neurotransmission through neurotrophins such as brain-derived neurotrophic factors (BDNF) involved in the reward circuitry, the dysregulation of which are implicated in the development of addiction [21,23]. Studies in chronic schizophrenia patients have shown decreased levels of BDNF [24,25]. Zhang et al. [26] studied the relationship between ND and severity of symptoms in subjects with schizophrenia and reported significantly higher levels of BDNF in smokers than in non-smokers. A negative correlation was also noted between BDNF levels and negative symptoms among those who smoked more cigarettes. These studies further support the notion that nicotine and other chemicals in cigarettes can affect the symptoms of mental illness and reinforce smoking in this group [21].

It is clear that people with mental illness have a shorter lifespan (10–25 years) than the general population [27] and smoking contributes to additional risk of mortality and morbidity in this population [28,29]. These reports add further evidence that smoking can be detrimental to people with mental illness, especially those with depression and psychosis. Although treatable, tobacco use is often overlooked due to the misconception that cessation might destabilise the mental condition of the patients [30]. Taylor et al. [31] had addressed this concern and concluded in their systematic review that there is enough evidence to show that smoking cessation improves quality of life (QoL) and mood while reducing symptoms of anxiety and depression. Smoking cessation is thus beneficial for people with mental illness and it is achievable as many of the mental health patients who smoke do express interest in quitting smoking [32]. 

Previous local studies have looked at the national population sample [11,12] and it is not clear how different the population with mental illness is as compared to the general public in terms of prevalence rates and smoking characteristics. Hence it is important to understand the smoking patterns, severity, and motivators/deterrents for cessation in the psychiatric population. This approach will help us to identify the factors (sociodemographic and clinical) that are associated with smoking and to identify the groups who are at higher risk of smoking but less likely to quit. Clinicians can identify such groups and plan the smoking cessation programs to meet the needs of these clients. Additionally, the motivators and deterrents endorsed by people with mental illness can also be taken into account while planning their smoking cessation and follow up. This approach will not only facilitate better smoking cessation outcomes but also improve the QoL and treatment outcomes of the individual and also contribute to better public health by reducing the mortality and morbidity among those with mental illness. 

This study assessed the prevalence and correlates of smoking among treatment-seeking psychiatric patients. It also examined the predictors of smoking and awareness towards ongoing cessation programmes to better understand the avenues for smoking cessation in this population. We hypothesise that (a) prevalence of smoking and ND among those with mental illness will be higher than that in the general population of Singapore, (b) smoking status will be associated with sociodemographic correlates such as age, gender, and ethnicity similar to the general population in Singapore, (c) awareness of institutional programmes of smoking cessation will be low and d) specific motivators/deterrents exist that can be incorporated into the cessation programmes. The results will not only allow clinicians to identify the risk groups but also refer them for suitable cessation programmes that will improve their health outcomes and quality of life. 

## 2. Materials and Methods 

### 2.1. Participants

Participants were recruited from the inpatient wards or outpatient clinics in Institute of Mental Health (IMH), a tertiary psychiatric care hospital in Singapore through (a) referral by the attending clinician (b) self-referral, in response to the recruitment posters displayed at the clinics. A trained study team member screened the participants to ensure that the inclusion criteria were met. Participants were included in the study if they were 21 to 65 years old and had a diagnosis of either depressive disorder or schizophrenia spectrum and other psychotic disorders. The study excluded participants who did not have the mental capacity to consent to the study. The attending clinician referred clinically stable patients who fulfilled the criteria to the study team members who screened them by verifying the eligibility against their medical records after obtaining the written consent. For participants who responded to the poster, eligibility was assessed by the trained study team members by checking the medical records. The trained study team members ensured that all the subjects were cognitively capable of understanding the consent and the survey questions. The survey was administered in either one of the four local languages: English, Chinese, Malay, or Tamil, as preferred by the participants. Translations were done by professional translation firms. Ethics approval was obtained from the Institutional Research Review Board and Domain Specific Review Board (Ref: 2018/00772) and written consent was taken from all the participants. 

### 2.2. Measures

#### 2.2.1. Smoking Habits and Cessation

A modified version of Global Adult Tobacco Survey (GATS [33]) was used to capture data that included sociodemographic information (age, gender, ethnicity, education, housing, income, etc.), and questions regarding their smoking status. The classification of smoker, non-smoker, and past smoker was based on the definitions from the national health interview survey where participants who had smoked at least 100 cigarettes in their lifetime and were currently smoking at the time of the survey were classified as a smoker. Participants who had smoked at least 100 cigarettes but had quit smoking at the time of the survey were classified as former smokers. Those who had never smoked or had smoked less than 100 cigarettes in their lifetime were classified as non-smokers [34]. The questionnaire also captured data on current and past smoking patterns and frequency, including questions on smoking habits where participants were asked if they smoke/used to smoke daily or less than daily (a few days of a week, a month, or a year). The branching of the questionnaire followed the GATS questionnaire (GATSv2, 2010) [33]. Frequency of smoking (daily or weekly), type of tobacco products used and their age of onset for tobacco use were captured for both current and past smokers. Questions also included the smoking status of their close family members (e.g., parents, siblings, and grandparents). Participants were also asked if they had used electronic nicotine delivery systems (ENDS), and the type, frequency and reasons for use. Smoking cessation and attitudes towards cessation were also assessed using the modified smoking cessation module of the GATS.

#### 2.2.2. Health Status

The clinical diagnosis of the participant was captured as indicated in the electronic medical records which follows the fourth edition of the Diagnostic and Statistical Manual of Mental Disorders (DSM-lV) criteria. Participants were also asked if their smoking started before their psychiatric condition was diagnosed and if they suffer/have suffered from any of the smoking related diseases. 

#### 2.2.3. Nicotine Dependence (ND)

Physiological dependence to nicotine was measured using the Fagerstrom Test for Nicotine Dependence (FTND) in smokers and past smokers [35,36]. FTND is a 6-item scale scored from 0 to 3. The total score is calculated by totalling the responses for the 6 items and a score of 0 to 4 is classified as low dependence, 5 to 7 as moderate and above 8 is scored as high dependence [12]. A cut-off score of 5 and above was set for nicotine dependence.

### 2.3. Analysis

#### 2.3.1. Sampling and Sample Size

The sample size for the study was initially calculated based on the data from Lasser et al. [17] to estimate the prevalence of current smoking for respondents with lifetime mental illness (34.8%). Based on this estimate, a target sample of 349 patients was required to provide sufficient precision to establish the prevalence of current smoking among this population. With the expectation of approximately 10% of missing data or partial completion, a total sample size of 384 was desirable for this study. A quota sampling was used based on gender, age and clinical diagnosis in order to get almost equal representation across these groups (40 to 60%) to match the patient population. Convenience sampling was used to recruit the participants for each quota.

#### 2.3.2. Statistical Analysis

Descriptive statistics was used to describe the sociodemographic factors, smoking habits, and cessation-related variables. Mean and standard errors were calculated for continuous variables and frequencies and percentages for categorical variables. Chi-square test was used to study if the sociodemographic variables and ND between the groups were significantly different. Multiple logistic regression was used to explore the risk factors of smoking. Age, gender, ethnicity, education, employment, income, diagnosis, and risk perception (assessed based on the question “Do you believe that breathing in smoke (passive smoking) can cause serious illness?”) were used as covariates in the analysis. All statistical analyses were carried out using SPSS (IBM Corp. Released 2017. IBM SPSS statistics for Windows, Version 25.0, Armonk, NY, USA.), with two-sided tests and *p*-value ≤ 0.05 being considered as statistically significant.

## 3. Results

### 3.1. Sociodemographics

A total of 411 subjects were screened and 380 participants took part in the study. The reasons for screen failures included diagnosis other than depressive disorder and schizophrenia spectrum and other psychotic disorder, lack of mental capacity, intellectual disability, etc. Table 1 shows the sociodemographic characteristics of the participants. Majority of the participants were male (55.3%), Chinese (73.4%), and single (69.5%). Nearly half of the participants (47.1%) were employed and the majority of the participants had no income or monthly income of less than $2000 (81.1%). The mean age of participants was 39.8 years (±12; median 41 years, range 21–65 years) and the majority of participants had a diagnosis of schizophrenia spectrum and other psychotic disorders (53.4%). There were statistically significant group differences for age, gender, ethnicity, marital status, education, income and diagnosis between smokers, past smokers, and non-smokers. A higher number of smokers belonged to the age group of 41 to 65 years compared to non-smokers and past smokers. The highest educational qualification for majority of the smokers was secondary or primary school whilst the past smokers and non-smokers were mostly pre-university/diploma holders. The majority of the smokers were unemployed compared to past smokers or non-smokers who were employed.

### 3.2. Prevalence of Smoking and Smoking Habits Subsection

The prevalence of smoking was 39.5% (*n* = 150) in the sample. Majority of the smokers were males (77.3%). Eighty-six percent of current smokers (*n* = 129) and 79.4% (*n* = 27) of past smokers were daily smokers. The mean age of smoking initiation was 16.4 years (SD ± 7.6) for current smokers and 16.5 years (±4.6) for past smokers. A family history of smoking was reported by 79.9% current smokers (*n* = 119) and 85.3% (*n* = 29) of past smokers. A large proportion of the smokers (88.7%) and past smokers (97%) reported onset of smoking before their mental illness was diagnosed (mean number of months: 137.8 ± 108; median: 108, range 0.75–480).

Cigarettes (91.3%, *n* = 137), followed by hand rolled cigarettes (40.7%, *n* = 61) were more commonly used by smokers and past smokers (100% and 23.5%). Current and lifetime use of ENDS was generally higher among past smokers than current smokers (current use 2.7% vs. 29.4%; lifetime use 28% vs. 41.2%), however, this difference was not statistically significant. Those who had used ENDS used it both locally and when they were overseas (*n* = 42). More than three quarters (*n* = 36) of those who reported lifetime use, used ENDs locally. The main reasons for not trying ENDS cited by current smokers included local availability (37.9%), higher cost (26.8%), and health concerns (18.5%). Among the 16 past smokers who had never used ENDS, 7 cited local availability, 5 were concerned about health risks and 2 felt that it is expensive. Nearly 15% (*n* = 16) of the smokers and 35% (*n* = 7) of past smokers had never heard of ENDS previously. 

### 3.3. Nicotine Dependence

Fifty-two percent of the current smokers (*n* = 78) and 30.3% (*n* = 10) of the past smokers had ND. The percentage of participants with ND was significantly higher in smokers than in past smokers (*p* = 0.022). There was no significant difference in the percentage of participants with ND between the smokers and past smokers across specific categories measured (low, moderate, high; *p* = 0.065, Table 2).

### 3.4. Factors Associated with Smoking

Factors including sociodemographic correlates (gender, ethnicity, and education), risk perception and diagnosis were found to be significantly associated with smoking. Females compared to males were less likely to smoke. Those of Malay and Indian ethnicities were more likely to smoke as compared to those of Chinese ethnicity. Those with primary education as the highest educational qualification had a higher likelihood of smoking than those with university education. Those with a diagnosis of schizophrenia spectrum and other psychotic disorders were less likely to smoke than those with depressive disorders. Those who thought that smoking can cause health problems were less likely to smoke than those who did not perceive a risk (Table 3).

### 3.5. Smoking Cessation: Motivators and Help Seeking

#### 3.5.1. Help Seeking in Smokers

**Figure 1:**
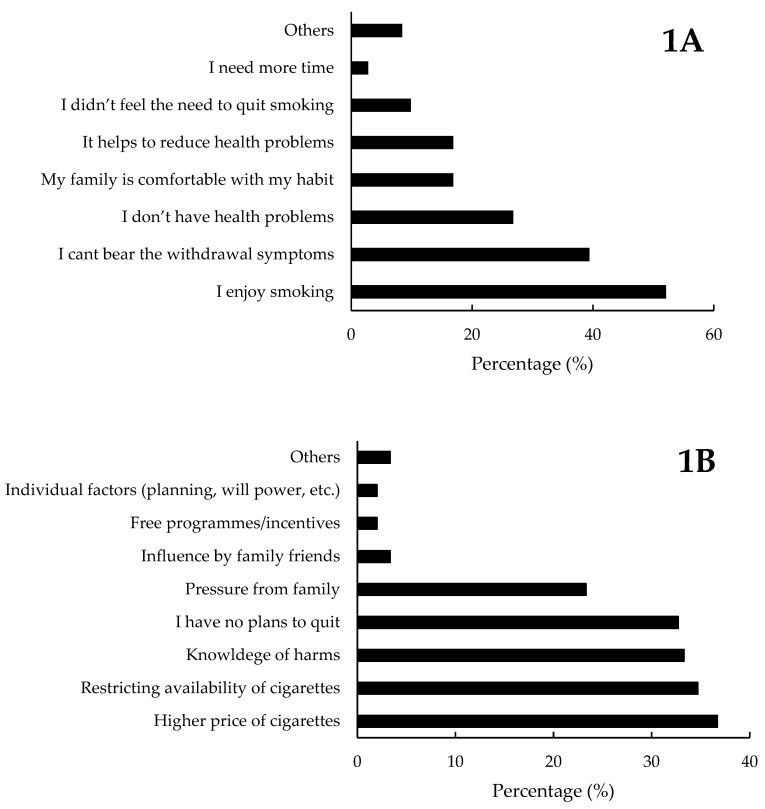
The common reasons cited by current smokers for not having any attempts to quit smoking in the past 12 months (**1A**) and factors that they felt will encourage smoking cessation (**1B**).

Half of the current smokers (52%, *n* = 78) had made at least one attempt to quit in the past 12 months. The top three reasons endorsed by the current smokers included the fact that a) they enjoyed smoking (52%), b) they cannot bear the withdrawal symptoms if they stop smoking (39.4%) and c) not having any health issues (26.8%). The detailed description is provided in Figure 1A. The reason for not considering smoking cessation was explored among those who did not report any attempts in the past 12 months. Eighty-six percent (*n* = 129) of the smokers had never sought medical help for smoking cessation in the past 12 months. The most common reasons cited by those who did not seek medical help for smoking cessation (*n* = 129) were a) no interest to quit smoking (48.8%), b) lack of awareness regarding the service (27.9%) and c) the self-reported confidence that they could quit whenever they want (27.1%). A small subset of the current smokers (14%, *n* = 21) employed counselling/cessation clinics (*n* = 10), nicotine replacement therapy (*n* = 10), tele-support (*n* = 2) and pharmacological methods (*n* = 1) in the past 12 months in their attempts to quit smoking. 

Although 44% (*n* = 66) of the smokers were aware of the institutional smoking cessation programmes tailored for the psychiatric population, only 4% (*n* = 6) of the smokers (*n* = 150) were enrolled in the programme during the time of the interview. Nearly half of the smokers (49.7%, *n* = 74) were willing to make use of the programme upon referral. The common reasons cited by those who refused referral included (a) not interested to quit (56.4%), (b) unsure about the cost and efficiency of the programme (20%) and (c) did not consider cessation as a priority at the time of interview (14.5%).

#### 3.5.2. Motivation to Quit Smoking in Current Smokers

More than half (56.7%, Figure 1B) of the current smokers had no plans to quit smoking in the next 6 months whereas, 32.7% had plans to quit in 6 months and 10.7% had plans to quit in one month (10.7%). Smokers were asked to endorse the factors that would encourage them to quit smoking. The top three factors included (a) higher price of cigarettes (36.7%), (b) restricting the availability of cigarettes (34.7%), and (c) increased knowledge of health-related harm (33.3%). 

#### 3.5.3. Help Seeking for Cessation in Past Smokers

Majority of the past smokers had been abstinent from nicotine for years (76.5%, *n* = 26) at the time of the interviews with a mean of 95.6 months (±112.6) since the cessation. The mean age at which they quit smoking successfully was 31.4 years (±11.3). Around 35% (*n* = 12) had one to two quit attempts, 32.4% (*n* = 11) had three to four and 23.5% (*n* = 8) had more than six attempts before successful cessation. Only four participants sought medical help for cessation and all of them used Nicotine Replacement Therapy (NRT) along with either tele-support, counselling, pharmacological methods, or traditional medicine. 

#### 3.5.4. Motivators and Other Factors in Successful Smoking Cessation: Past Smokers

**Figure 2:**
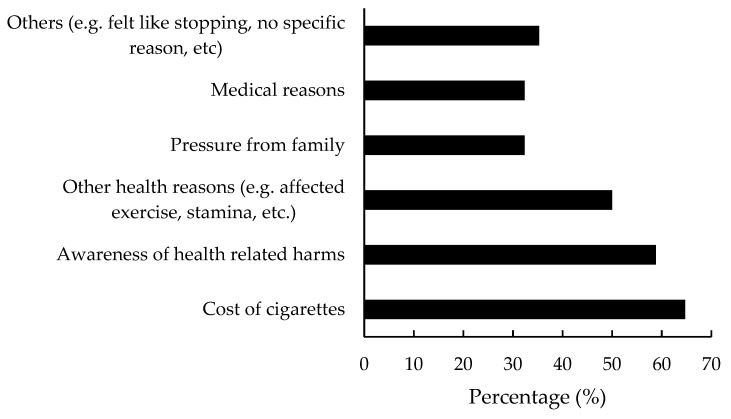
Factors that encouraged the past smokers to successfully quit smoking.

The factors that motivated them to stop smoking included the cost of cigarettes (64.7%), awareness of health-related harms (58.8%), and other health related reasons such as problems with exercise routines, loss of strength and stamina, etc. (50%, Figure 2). Participants reported that the important factor/method that helped them to achieve successful smoking cessation was their own will-power/self-determination (51.4%), substitution with other substances such as sweets, snacks, coffee, e-cigarettes, etc. (14.2%), and other medical interventions (NRT, counselling, etc. 11.4%).

## 4. Discussion

The study found that the prevalence of current smoking was 39.5% among the psychiatric population, which was 2.4 times higher than that in the general population of Singapore. This is consistent with previous studies where higher prevalence of smoking was noted among people with mental illness [18,37,38]. Smith et al. [18] reported a similar smoking prevalence rate (39%), where patients with a diagnosis of mental illness showed 3.2 times higher odds of current smoking than those without a diagnosis. Those with a diagnosis of mental illness were 25% less likely to quit smoking, which has been suggested to be due to a lower motivation to quit. A lower readiness to quit (56.7%) was observed among the current smokers in our study. Siru et al. [39] reviewed the literature and compared the motivation to quit smoking among people with mental illness with that of the general population and concluded that people with mental illness are as motivated as the general population to quit smoking. However, the authors noted that those with psychotic conditions are less motivated than those with depression thus showing a difference in motivation among different mental disorders. Our quota sampling allowed almost half of the participants to have either a diagnosis of depressive disorder or schizophrenia spectrum and other psychotic disorders, which could explain the lower readiness to quit reported in the current sample. 

Cigarettes are the most easily available tobacco product locally due to the policy regulations restrict the sale of other tobacco products such as ENDS, which are not legal in Singapore. Nonetheless, some participants reported the use of ENDS. We also observed that past smokers used ENDS more frequently than current smokers (lifetime use; 41.2% vs. 28%; current use/use when actively smoking (for past smokers): 29.4% vs. 2.7%). This is in contrast with the literature, where current smokers are more likely to use ENDS than past smokers [40,41]. This discrepancy could be explained based on the findings by Richardson et al. [42] who observed that people who are considering quitting tend to use ENDS more to aid in smoking cessation. However, the use of ENDS did not favour successful cessation [42] which is in agreement with our current results where ENDS was not endorsed by the past smokers as a method that had helped them to stop smoking. Despite the ban in Singapore, which prohibits the sale and use of ENDS, the lifetime use of ENDS was comparable with international data [42] with participants reporting both local and overseas use of ENDS. 

ND was seven times higher in the study sample than in the general population (23.2% vs. 3.3%) [12]. Previous studies have reported higher ND among people with mental illness [18,37,38]. We also observed a significant difference in ND between current smokers and past smokers. Current smokers had a significantly higher rate of ND than past smokers. While a higher proportion of past smokers showed low dependence/no dependence, there were more smokers with moderate and high dependence. This could be a possible reason for the successful smoking cessation observed among past smokers, and also suggests that those with higher severity of ND need additional interventions for successful cessation. Similar results are reported by other studies, further confirming the need for additional resources to aid cessation in current smokers with higher ND [36,43].

Smoking is used as a coping mechanism to deal with stress [44]. While some studies support smoking initiation as a coping mechanism for subjects with mental illness [45], others contradict the findings to show that smoking precedes mental illness [46,47]. Our data shows that smoking preceded the clinical diagnosis of mental illness with 88% of the participants reporting smoking initiation 9 years (median 9 years; mean: 11 years) earlier than their diagnosis. It is not clear if the participants were experiencing symptoms of mental illness at the time of smoking initiation and smoking was used as a coping mechanism to deal with the symptoms of the disease. A detailed investigation on the factors surrounding smoking initiation could answer this question. This is an important research area that should be explored in the future research studies.

We observed significant inter-group differences in demographic characteristics, an observation that was previously noted in national adult surveys [43,48]. Sociodemographic factors (gender, education and ethnicity) were associated with smoking in the study sample. The findings of our study are consistent with previous reports where males, those with primary education, a diagnosis of depression, and lower risk perception, are more likely to smoke [49,50]. People with depressive symptoms may smoke more as a coping mechanism to deal with the symptoms of depression [51] and therefore have a lower possibility of successfully quitting, even if they are motivated [52]. Therefore, this specific sociodemographic group who is at a higher risk of smoking and thus smoking-related complications should be given special attention and interventions to improve the cessation rates.

Our study showed that around 52% of the smokers had made at least one attempt to quit smoking in the past 12 months. Hymowitz et al. [53] have shown that 67% of the smokers had made at least one serious attempt to quit, supporting our observations. The reasons given for quitting were health concerns, expense, and concerns regarding exposing people to passive smoke. See et al. [43] studied an inpatient population in Singapore who sought treatment for smoking cessation and noted that cost, social pressure and health concerns were major motivators for smoking cessation. Similar reasons were given by the past smokers in the current study where expense and health risks were highlighted as major motivators among smokers and past smokers. Only a small proportion of the smokers and past smokers sought help for smoking cessation, which is also reported by other studies [54]. The reasons cited by the participants of this study included lack of awareness regarding the service and having no intention to quit. Our study showed that only 44% of the smokers were aware of the institutional smoking cessation programmes for psychiatric patients and only 4% were enrolled in the programmes at the time of the interview. Singapore has one of the most effective strategies for treatment of tobacco dependence with a range of smoking cessation services embedded in the community, schools, and workplaces [55]. Healthcare institutions have cessation clinics to integrate smoking cessation with clinical management, especially for the vulnerable groups. The hospital based smoking cessation programmes in Singapore include pharmacotherapy and behavioural therapy (counselling and tele-support) with an intent to achieve 20% to 36% quit rates [43,55]. The treatment is patient specific, based on their medical history, sociodemographic factors, lifestyle factors and psychiatric conditions. The institutional programmes in IMH are thus developed specifically for a psychiatric population and includes pharmacotherapy, stress management, lifestyle changes, and counselling tailored for the patient’s needs [56]. Patients are followed up regularly by the attending clinicians, with a goal of 50% reduction in smoking between appointments. While a lot of effort and resources are being spent for smoking cessation programmes, the awareness creation among potential beneficiaries are often overlooked and thus the people who are in need of the services are not aware of the services available and do not benefit from it. 

The participants had endorsed that the higher cost of cigarettes, restricting availability, and awareness regarding harms could discourage smoking. Those who successfully quit smoking in our study agreed that the cost of cigarettes was one of the crucial factors for their decision to quit. Singapore has a stringent tobacco control programme which includes key elements such as restricting the sale of tobacco products, controlling advertisements, smoking prohibition at public places, customs regulation for importing tobacco products and educational initiatives to curtail tobacco use [55]. Singapore imposes comparatively higher tobacco taxes compared to other ASEAN countries, which was adjusted based on the national prevalence of smoking over the years. Nonetheless, the prevalence of smoking remains unchanged in the population, with no sign of reduction over the years [11,12]. In view of the endorsement of both smokers and past smokers, increasing the cost of cigarettes is one of the strong deterrents of smoking. Therefore, more stringent policies together with educational initiatives based on the local data could promote smoking cessation. 

This study has identified important sociodemographic and clinical factors that are associated with smoking. It has also identified the groups (males, lower education status, diagnosis of depression, lower risk perception, non-Chinese ethnicity) who are at higher risk of smoking but less likely to quit. It was also noted that the awareness towards institutional smoking cessation programmes was low, which could be a reason for the lower attendance in the smoking cessation clinic [56] despite the higher prevalence rate of smoking. Educational/awareness programmes that introduce them to institutional initiatives can improve the enrolment to the programmes, which in turn can enhance rates of cessation among these clients. Clinicians can identify these groups and plan the smoking cessation programs to meet the needs of these clients. Additionally, the motivators and deterrents endorsed by the psychiatric population can also be taken into account while planning their smoking cessation treatment and follow up. In Singapore, more than 2000 Singaporeans die from smoking related diseases every year [57] and approximately S$600 million is spent to manage the direct healthcare costs and economic costs due to lost productivity [58]. Smoking predisposes people with mental illness to other medical conditions [59] and they carry a substantial share of medical and social burden due to their heavy smoking [60]. Nearly half of the deaths among hospitalised patients with mental illness were shown to be due to smoking related diseases [60]. Considering the higher prevalence of smoking and smoking related mortality and morbidity in this group, promoting smoking cessation is imperative to reduce health burden. 

The limitations of the study include the small sample size for the past smokers, which made the comparisons between the groups challenging. The data is captured through self-report and included questions regarding age of initiation, smoking patterns and types of services used, which are subject to recall bias. The study used a quota sampling and thus care should be taken while generalising the results to the wider population.

## 5. Conclusions

Those with mental illness have a higher prevalence of smoking and ND than the general population, and a lower readiness to quit smoking. Specific sociodemographic characteristics were associated with current smoking. The findings from the study indicate that policy changes to restrict the availability of cigarettes, increase the cost and awareness creation towards the health-related risks could promote smoking cessation. While a lot of resources are directed towards cessation programmes, the accessibility of the programmes remains suboptimal and thus needs to be improved in order to reduce the health disparity between psychiatric patients and the general public. 

## Figures and Tables

**Table 1:** Socio-demographic characteristics of the participants.

Socio-Demographic Factors		Smokers *n* (%) *n* = 150	Past Smokers *n* (%) *n* = 34	Non-Smokers *n* (%) *n* = 196	Total	*p*-Value
Age	21–40	61(40.7)	18(52.9)	109 (55.6)	188 (49.5)	0.02
	41–65	89 (59.3)	16 (47.1)	87(44.4)	192 (50.5)	
Gender	Male	116 (77.3)	23 (67.6)	71(36.2)	210 (55.3)	<0.001
	Female	34 (22.7)	11(32.4)	125 (63.8)	170 (44.7)	
Ethnicity	Chinese	92 (61.3)	25 (73.5)	162 (82.7)	279 (73.4)	0.002
	Malay	32 (21.3)	5 (14.7)	18 (9.2)	55 (14.5)	
	Indian	21(14.0)	4 (11.8)	14 (7.1)	39 (10.3)	
	Others	5 (3.3)	0	2 (1.0)	7 (1.8)	
Marital status	Married	21(14.0)	9 (26.5)	32 (16.3)	62 (16.3)	0.001
	Single	94 (62.7)	22 (64.7)	148 (75.5)	264 (69.5)	
	Divorced/separated/widowed	10 (18.7)	0	14 (8.1)	54 (13.2)	
Nationality	Singaporean	146 (97.3)	32 (94.1)	190 (96.9)	368 (96.8)	0.662
	Permanent Resident	4 (2.7)	2 (5.9)	6(3.1)	12 (3.2)	
Education	Primary or lower	34 (22.7)	4 (11.8)	13 (6.6)	51(13.4)	<0.001
	Secondary	47(31.3)	9(26.5)	46 (23.5)	102 (26.8)	
	Pre-U/diploma	28 (18.7)	15 (44.1)	69 (35.2)	112 (29.5)	
	Vocational/ITE	26 (17.3)	2 (5.9)	25 (12.8)	53 (14)	
	Degree and above	15 (10.0)	4 (11.8)	43 (21.9)	62 (16.3)	
Employment	Employed	60 (40.0)	21(61.8)	98 (50.0)	179 (47.1)	0.064
	Unemployed	78 (52)	11(32.4)	77(39.3)	166 (43.7)	
	Economically inactive	12 (8.0)	2 (5.9)	21(10.7)	35 (10.7)	
Income	Below 2000	131(87.3)	23 (67.6)	154 (78.6)	308 (81.1)	0.02
	2000 to 3999	17(11.3)	7 (20.6)	30 (15.3)	54 (14.2)	
	4000 and above	2 (1.3)	4 (11.8)	12 (6.1)	18 (4.7)	
Children	Yes	39 (26.2)	11(32.4)	34 (17.3)	84 (22.2)	0.048
	No	110 (73.8)	23 (67.6)	162 (82.7)	295 (77.8)	
Mean number of household members		3.25 (±2.1)	3.32 (±1.12)	3.26 (±1.31)	3.26 (±1.64)	0.973
Diagnosis	Depressive disorder	73 (48.7)	22 (64.7)	82 (41.8)	177 (46.6)	0.038
	Schizophrenia spectrum and other psychotic disorder	77 (51.3)	12 (35.3)	114 (58.2)	203 (53.4)	

**Table 2:** Nicotine dependence among smokers and past smokers.

Dependence	Smokers *n* (%)	Past Smokers *n* (%)
Low (0–4)	71(47.7)	23 (69.7)
Moderate (5–7)	56(37.6)	8 (24.2)
High (8 and above)	22 (14.8)	2 (6.1)
Overall (5 and above)	78 (52.3%)	10 (30.3)

**Table 3:** Factors associated with ever smoking among psychiatric patients.

Variables		Odds Ratio	CI (95%)	*p*-Value
Age	21–40	Ref.		
	41–65	1.6	0.8–3.1	0.152
Gender	Male	Ref.		
	Female	0.1	0.1–0.2	0.000
Ethnicity	Chinese	Ref.		
	Malay	3.8	1.7–8.6	0.001
	Indian	3.0	1.2–7.4	0.016
	Others	16.4	1.2–233.6	0.039
Marital status	Married	Ref.		
	Single	1.3	0.5–3.5	0.559
	Divorced/separated/widowed	2.4	0.8–7.4	0.134
Education	Degree and above	Ref.		
	Primary or lower	6.1	1.8–21.1	0.004
	Secondary	2.4	0.9–6.1	0.069
	Pre-U/diploma	1.1	0.5–2.5	0.829
	Vocational/ITE	1.2	0.4–3.4	0.702
Employment	Employed	Ref.		
	Unemployed	0.9	0.5–1.6	0.665
	Economically inactive	0.6	0.2–1.5	0.269
Income	Below 2000	Ref.		
	2000 to 3999	0.7	0.3–1.7	0.444
	4000 and above	1.2	0.3-4.7	0.766
Children	No	Ref.		
	Yes	0.7	0.2–1.7	0.395
Mean number of household members		1.0	0.9–1.3	0.635
Diagnosis	Depressive disorder	Ref.		
	Schizophrenia spectrum and other psychotic disorders	0.3	0.2–0.6	0.000
Risk perception	Yes	Ref.		
	No	7.9	2.3–27.9	0.001

## References

[1] World Health Organization (WHO) (2019). WHO Launches New Report on Global Tobacco Use Trends. https://www.who.int/news-room/detail/19-12-2019-who-launches-new-report-on-global-tobacco-use-trends.

[2] Banks E., Joshy G., Weber M.F., Liu B., Grenfell R., Egger S., Paige E., Lopez A.D., Sitas F., Beral V. (2015). Tobacco smoking and all-cause mortality in a large Australian cohort study: Findings from a mature epidemic with current low smoking prevalence. BMC Med..

[3] Shah S.R., Cole J.W. (2010). Smoking and stroke: The more you smoke the more you stroke. Expert Rev. Cardiovasc. Ther..

[4] Jethwa A.R., Khariwala S.S. (2017). Tobacco-related carcinogenesis in head and neck cancer. Cancer Metastasis Rev..

[5] World Health Organization WHO (2005). Framework Convention on Tobacco Control.

[6] Zheng W., McLerran D.F., Rolland B.A., Fu Z., Boffetta P., He J., Gupta P.C., Ramadas K., Tsugane S., Irie F. (2014). Burden of Total and Cause-Specific Mortality Related to Tobacco Smoking among Adults Aged ≥45 Years in Asia: A Pooled Analysis of 21 Cohorts. PLoS Med..

[7] Reitsma M.B., Fullman N., Ng M., Salama J.S., Abajobir A.A., Abate K.H., Abbafati C., Abera S.F., Abraham B., Abyu G.Y. (2017). Smoking prevalence and attributable disease burden in 195 countries and territories, 1990–2015: A systematic analysis from the Global Burden of Disease Study 2015. Lancet.

[8] Katanoda K., Jiang Y., Park S., Lim M.K., Qiao Y.-L., Inoue M. (2014). Tobacco control challenges in East Asia: Proposals for change in the world’s largest epidemic region. Tob. Control.

[9] Ministry of Health National Health Survey. https://www.moh.gov.sg/content/dam/moh_web/Publications/Reports/2011/NHS2010%20-%20low%20res.pdf.

[10] (2017). National Population Health Survey. https://www.moh.gov.sg/docs/librariesprovider5/resources-statistics/reports/executive-summary-nphs-2016_17.pdf.

[11] Picco L., Subramaniam S., Abdin E., Vaignakar J.A., Chong S.A. (2012). Smoking and nicotine dependence in Singapore: Finding from a Cross-Sectional Epidemiology study. Ann. Acad. Med. Singap..

[12] Shahwan S., Abdin E., Shafie S., Chang S., Sambasivam R., Zhang Y., Vaingankar J.A., Teo Y.Y., Heng D., Chong S.A. (2019). Prevalence and correlates of smoking and nicotine dependence: Results of a nationwide cross-sectional survey among Singapore residents. BMJ Open.

[13] National Registry of Disease Office Publications, Annual Report. https://www.nrdo.gov.sg/publications.

[14] Dierker L.C., Ramirez R.R., Chavez L.M., Canino G. (2005). Association between psychiatric disorders and smoking stages among Latino adolescents. Drug Alcohol Depend..

[15] Wilens T., Biederman J., Adamson J., Henin A., Sgambati S., Gignac M., Sawtelle R., Santry A., Monuteaux M.C. (2008). Further evidence of an association between adolescent bipolar disorder with smoking and substance use disorders: A controlled study. Drug Alcohol Depend..

[16] Grant B.F., Hasin D.S., Chou S.P., Stinson F.S., Dawson D.A. (2004). Nicotine dependence and psychiatric disorders in the United States: Results from the national epidemiologic survey on alcohol and related conditions. Arch. Gen. Psychiatry.

[17] Lasser K., Boyd J.W., Woolhandler S., Himmelstein D.U., McCormick D., Bor D.H. (2000). Smoking and mental illness: A population-based prevalence study. J. Am. Med. Assoc..

[18] Smith P.H., Mazure C.M., McKee S.A. (2014). Smoking and mental illness in the US population. Tob. Control.

[19] Li X.-H., An F.-R., Ungvari G.S., Ng C.H., Chiu H.F.K., Wu P.-P., Jin X., Ning Y. (2017). Prevalence of smoking in patients with bipolar disorder, major depressive disorder and schizophrenia and their relationships with quality of life. Sci. Rep..

[20] Wootton R.E., Richmond R.C., Stuijfzand B.G., Lawn R.B., Sallis H.M., Taylor G.M., Hemani G., Jones H.J., Zammit S., Smith G.D. (2019). Evidence for causal effects of lifetime smoking on risk for depression and schizophrenia: A Mendelian randomisation study. Psychol. Med..

[21] Ziedonis D., Williams J.M., Smelson D. (2003). Serious Mental Illness and Tobacco Addiction: A Model Program to Address This Common but Neglected Issue. Am. J. Med. Sci..

[22] Winterer G. (2010). Why do patients with schizophrenia smoke?. Curr. Opin. Psychiatry.

[23] Kenny P.J., File S.E., Rattray M. (2000). Acute nicotine decreases, and chronic nicotine increases the expression of brain-derived neuro- trophic factor mRNA in rat hippocampus. Brain Res. Mol. Brain Res..

[24] Rizos E.N., Rontos I., Laskos E., Arsenis G., Michalopoulou P.G., Vasilopoulos D., Gournellis R., Lykouras L. (2008). Investigation of serum BDNF levels in drug-naive patients with schizophre-nia. Prog. Neuropsychopharmacol. Biol. Psychiatry.

[25] Xiu M.H., Hui L., Dang Y.F., Hou T.D., Zhang C.X., Zheng Y.L., Chen D.C., Kosten T.R., Zhang X.Y. (2009). Decreased serum BDNF levels in chronic institutionalized schizophrenia on long-term treatment with typical and atypical antipsychotics. Prog. Neuropsychopharmacol. Biol. Psychiatry.

[26] Zhang X.Y., Xiu M.H., De Yang F., Wu G.Y., Lu L., Kosten T.A., Kosten T.R. (2010). Nicotine dependence and serum BDNF levels in male patients with schizophrenia. Psychopharmacology.

[27] World Health Organization (2017). Premature Death among People with Severe Mental Disorders. https://www.who.int/mental_health/management/info_sheet.pdf.

[28] Bandiera F.C., Anteneh B., Le T., Delucchi K., Guydish J. (2015). Tobacco-related mortality among persons with mental health and substance abuse problems. PLoS ONE.

[29] Cheng K.Y., Chen S.Y. (2019). Avoidable mortality among long-stay patients with schizophrenia under different smoking-restriction settings. Taiwan J. Psychiatry.

[30] McNally L., Oyefeso A., Annan J., Perryman K., Bloor R., Freeman S., Wain B., Andrews H., Grimmer M., Crisp A. (2006). A survey of staff attitudes to smoking-related policy and intervention in psychiatric and general health care settings. J. Public Health.

[31] Taylor G., McNeill A., Girling A., Farley A., Lindson-Hawley N., Aveyard P. (2014). Change in mental health after smoking cessation: Systematic review and meta-analysis. Bmj.

[32] Stockings E., Bowman J., McElwaine K., Baker A., Terry M., Clancy R., Bartlem K., Wye P., Bridge P., Knight J. (2013). Readiness to quit smoking and quit attempts among Australian mental health inpatients. Nicotine Tob. Res..

[33] World Health Organization (2015). Global Adult Tobacco Survey. https://www.who.int/tobacco/publications/surveillance/gatstlas/en/.

[34] Centre for Disease Control, National Health Interview Survey. https://www.cdc.gov/nchs/nhis/tobacco/tobacco_glossary.htm.

[35] Fagerström K., Furberg H. (2008). A comparison of the Fagerström Test for Nicotine Dependence and smoking prevalence across countries. Addiction.

[36] Fagerström K.O., Kunze M., Schoberberger R., Breslau N., Hughes J.R., Hurt R.D., Puska P., Ramstrom L., Zatonski W. (1996). Nicotine dependence versus smoking prevalence: Comparisons among countries and categories of smokers. Tob. Control.

[37] De Leon J., Becoña E., Gurpegui M., Gonzalez-Pinto A., Diaz F.J. (2002). The Association between High Nicotine Dependence and Severe Mental Illness May Be Consistent across Countries. J. Clin. Psychiatry.

[38] Hughes J.R., Hatsukami D.K., Mitchell J.E., Dahlgren L.A. (1986). Prevalence of Smoking among Psychiatric Outpatients. Am. J. Psychiatry.

[39] Siru R., Hulse G.K., Tait R.J. (2009). Assessing Motivation to Quit Smoking in People with Mental Illness: A Review. Addiction.

[40] Caraballo R.S., Jamal A., Nguyen K.H., Kuiper N.M., Arrazola R.A. (2016). Electronic Nicotine Delivery System Use among U.S. Adults, 2014. Am. J. Prev. Med..

[41] Weaver S.R., Majeed B.A., Pechacek T.F., Nyman A.L., Gregory K.R., Eriksen M.P. (2016). Use of Electronic Nicotine Delivery Systems and Other Tobacco Products among USA Adults, 2014: Results from a National Survey. Int. J. Public Health.

[42] Richardson A., Pearson J., Xiao H., Stalgaitis C., Vallone D. (2014). Prevalence, Harm Perceptions, and Reasons for Using Noncombustible Tobacco Products among Current and Former Smokers. Am. J. Public Health.

[43] See J.H.J., Yong T.H., Poh S.L.K., Lum Y.C. (2019). Smoker motivations and predictors of smoking cessation: Lessons from an inpatient smoking cessation programme. Singap. Med. J..

[44] Siqueira L., Diab M., Bodian C., Rolnitzky L. (2000). Adolescents Becoming Smokers: The Roles of Stress and Coping Methods. J. Adolesc. Health.

[45] Baker T.B., Piper M.E., McCarthy D.E., Majeskie M.R., Fiore M.C. (2004). Addiction Motivation Reformulated: An Affective Processing Model of Negative Reinforcement. Psychol. Rev..

[46] Rohde P., Lewinsohn P.M., Brown R.A., Gau J.M., Kahler C.W. (2003). Psychiatric Disorders, Familial Factors and Cigarette Smoking: I. Associations with Smoking Initiation. Nicotine Tob. Res..

[47] Gurpegui M., Martínez-Ortega J.M., Aguilar M.C., Diaz F.J., Quintana H.M., de Leon J. (2005). Smoking Initiation and Schizophrenia: A Replication Study in a Spanish Sample. Schizophr. Res..

[48] Schauer G.L., Malarcher A.M., Berg C.J. (2014). Differences in Smoking and Cessation Characteristics among Adult Nondaily Smokers in the United States: Findings from the 2009–2010 National Adult Tobacco Survey. Nicotine Tob. Res..

[49] Hu L., Sekine M., Gaina A., Nasermoaddeli A., Kagamimori S. (2007). Association of Smoking Behavior and Socio-Demographic Factors, Work, Lifestyle and Mental Health of Japanese Civil Servants. J. Occup. Health.

[50] Kick S.D., Cooley D.D. (1997). Depressive, Not Anxiety, Symptoms Are Associated with Current Cigarette Smoking among University Internal Medical Patients. Psychosomatics.

[51] Escobedo L.G., Reddy M., Giovino G.A. (1998). The Relationship between Depressive Symptoms and Cigarette Smoking in US Adolescents. Addiction.

[52] Haukkala A., Uutela A., Vartiainen E., McAlister A., Knekt P. (2000). Depression and Smoking Cessation: The Role of Motivation and Self-Efficacy. Addict. Behav..

[53] Hymowitz N., Cummings K.M., Hyland A., Lynn W.R., Pechacek T.F., Hartwell T.D. (1997). Predictors of Smoking Cessation in a Cohort of Adult Smokers Followed for Five Years. Tob. Control.

[54] Ferguson S.G., Shiffman S., Gitchell J.G., Sembower M.A., West R. (2009). Unplanned Quit attempts—Results from a U.S. Sample of Smokers and Ex-Smokers. Nicotine Tob. Res..

[55] Amul G.G.H., Pang T. (2018). Progress in tobacco control in Singapore: Lessons and challenges in the implementation of the framework convention on tobacco control. Asia Pac. Policy Stud..

[56] Low L.T., Ng C.W., Lee C. (2019). Nicotine dependence treatment: Provision of a dedicated programme by the National Addictions Management Service. Singap. Med. J..

[57] Ministry of Health (2018). Proposed Tobacco Contro Measures in Singapore-The Standardized Packaging Proposal. Outcome of the 2018 Public Consultation and the Government’s Final Assessment. https://www.moh.gov.sg/docs/librariesprovider5/default-document-library/outcome-of-the-2018-public-consultation-and-the-government-s-final-assese3bf46dd03e8495998d07cdf5cde7e9f.pdf.

[58] Cher B.P., Chen C., Yoong J. (2017). Prevalence-based, disease-specific estimate of the social cost of smoking in Singapore. BMJ Open.

[59] Callaghan R.C., Veldhuizen S., Jeysingh T., Orlan C., Graham C., Kakouris G., Remington G., Gatley J. (2014). Patterns of tobacco-related mortality among individuals diagnosed with schizophrenia, bipolar disorder, or depression. J. Psychiatr. Res..

[60] Prochaska J.J., Das S., Young-Wolff K.C. (2017). Smoking, mental illness, and public health. Annu. Rev. Public Health.

